# Increased Mortality in Patients With Acutely Decompensated Heart Failure During the COVID-19 Pandemic in Toronto, Canada

**DOI:** 10.1016/j.cjco.2022.06.006

**Published:** 2022-06-22

**Authors:** Tayler A. Buchan, Lakshmi Kugathasan, Jeremy Kobulnik, Stephanie Poon, Kyle Runeckles, Steve Fan, Heather J. Ross, Ana C. Alba

**Affiliations:** a Peter Munk Cardiac Centre, University Health Network, Toronto, Ontario, Canada; b Division of Cardiology, Mount Sinai Hospital, Toronto, Ontario, Canada; c Division of Cardiology, Sunnybrook Health Sciences Centre, Toronto, Ontario, Canada

## Abstract

**Background:**

Coronavirus disease 2019 (COVID-19) has resulted in a reduction in patients seeking timely consultation for illnesses that are not related to COVID-19. Previously, we reported a decline in the number of emergency department (ED) visits and hospitalizations for acute decompensated heart failure (ADHF) during the 2020 COVID-19 pandemic vs that in 2019. We aimed to determine the consequences of these early trends on ADHF-patient morbidity and mortality.

**Methods:**

We compared consecutive patients presenting with ADHF to 3 academic medical centres in Toronto, Canada from March 1-September 28, 2020, vs those from the same time period in 2019. We used multivariate logistic regression models to evaluate whether the odds of hospitalization after presenting to the ED, recurrent ED visits or readmission within 30 days, and in-hospital all-cause mortality differed by timeframe.

**Results:**

We observed that, during the COVID-19 pandemic, a lower total number of patients presented to the hospital with ADHF, vs that in 2019. Despite this difference, the probability of being admitted to the hospital did not differ for patients seen in 2020 vs 2019. Among ADHF patients admitted to the hospital, however, we observed a significantly higher proportion being admitted to the intensive care unit, and a relative 66% increase in in-hospital mortality during the 2020 COVID-19 era, compared to that in 2019.

**Conclusions:**

Our findings suggest that improved messaging may be needed for patients living with chronic health conditions, including HF, during the pandemic, to educate and encourage them to present to hospital services when in need.

## Graphical abstract

**Figure:**
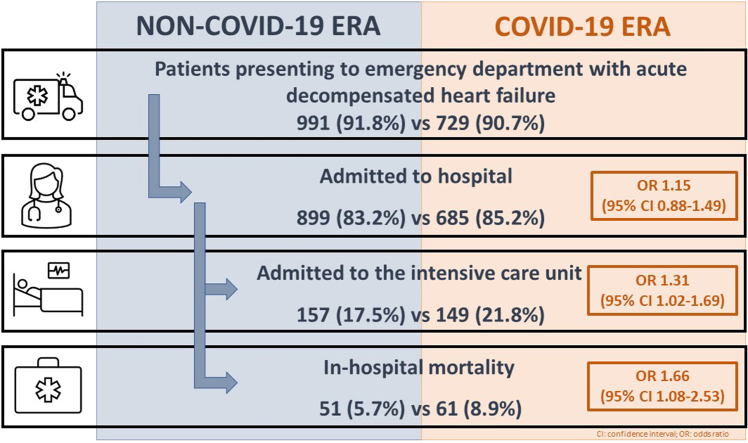

## Résumé

**Contexte:**

La maladie à coronavirus 2019 (COVID-19) s'est traduite par une diminution du nombre de patients demandant des consultations médicales pour des états de santé sans lien avec la COVID-19. Nous avons précédemment décrit une diminution du nombre de consultations aux urgences et d’hospitalisations en raison d’une insuffisance cardiaque aiguë décompensée (ICAD) au cours de la pandémie de COVID-19 en 2020, par rapport à 2019. Nous avons voulu déterminer les conséquences de ces tendances précoces sur la morbidité et la mortalité chez les patients atteints d’ICAD.

**Méthodologie:**

Nous avons comparé les données pour les patients consécutifs atteints d’ICAD de trois centres médicaux hospitaliers de Toronto (Canada) traités entre le 1^er^ mars et le 28 septembre 2020 et durant la même période en 2019. À l'aide de modèles de régression logistique multivariée, nous avons évalué les différences entre les probabilités d’hospitalisation après une consultation aux urgences, de consultations récurrentes aux urgences ou de réadmission dans les 30 jours suivant la visite initiale, ainsi que de mortalité hospitalière toutes causes confondues pour les patients vus durant ces deux périodes.

**Résultats:**

Durant la pandémie de COVID-19, le nombre total de patients atteints d’ICAD s’étant présentés à l’hôpital a été plus faible que celui relevé pour l’année 2019. Malgré cet écart, la probabilité d’admission à l’hôpital ne différait pas pour les patients vus en 2020 et en 2019. Parmi les patients atteints d’ICAD admis à l’hôpital, nous avons toutefois observé une proportion significativement plus élevée de séjours aux soins intensifs et une hausse relative de 66 % du taux de mortalité hospitalière, en comparant les données de 2020 (pandémie de COVID-19) et celles de 2019.

**Conclusions:**

Nos observations indiquent qu’il pourrait être nécessaire d’améliorer la communication avec les patients atteints de problèmes de santé chroniques (y compris l’IC) en situation de pandémie de façon à mieux les informer et à les encourager à consulter les services hospitaliers lorsque nécessaire.

Almost 2 years beyond the emergence of coronavirus disease 2019 (COVID-19), the world continues to battle the pandemic and a myriad of other collateral economic, societal, and health crises.^1^ Public health stay-at-home orders, physical distancing measures, growing amounts of cases, hospitalizations, ventilator-dependence, and deaths, as well as the subsequent reconfiguration of healthcare delivery have all had unintended consequences, including amplified patient fear and anxiety.^2^ This situation has led to a reduction in patients seeking timely consultations for illnesses that are not related to COVID-19.^3^

In 2020, during the early stages of the pandemic, we described the volume and characteristics of patients with acute decompensated heart failure (ADHF) presenting to the University Health Network (UHN), in Toronto, Canada, compared with those of a time-matched 2019 cohort.^4^ In this early study, we reported a decline in ADHF-related emergency department (ED) visits and hospitalizations. Unclear at the time was whether this decline reflected a tendency of patients to simply avoid the hospital setting, or whether changes in patient behavioural patterns combined with effective medical management via other platforms had circumvented the need for presentation at the hospital or for admission. Understanding the impact of the observed changes is critical, as it would highlight the need for either improved public health messaging, to reassure and educate patients on hospital safety measures and the importance of seeking timely medical attention, or adoption of newer patient-management strategies within routine practice. Therefore, to determine the consequences of the observed early trends on ADHF-patient morbidity and mortality, we sought to compare ADHF-related admission rates, the number of recurrent ED visits and readmissions within 30 days, and mortality, in consecutive patients presenting to 3 academic medical centres in Toronto, Canada from March 1 to September 28, 2020, vs those in the same time period in 2019.

## Methods

### Study design

In this multicentre cohort observational study, we compared consecutive patients presenting with ADHF to the UHN (Toronto General Hospital and Toronto Western Hospital), Mount Sinai Hospital (MSH), and Sunnybrook Health Sciences Centre (SHSC), in Toronto, Canada, from March 1 to September 28, 2020, and from March 1 to September 28, 2019 (time-matched control cohort). Public health messaging, including stay-at-home messaging, as well as adjustments made at cardiac clinics were initiated as of March 1, 2020, and this timeframe also contains the sentinel public lockdown date in mid-March within Ontario, Canada (Supplemental Fig. S1). This study was reviewed and approved by each of the institutional research ethics boards (REB #20-5326 [UHN], #20-0136-E [MSH], and #1955 [SHSC]).

### Study setting

At each of these 3 centres, rapid virtualization of cardiac care began in March 2020 (March 9, March 16, and March 27 at UHN, MSH, and SHSC, respectively), at which point in-person clinic appointments were rescheduled or replaced by videoconferencing visits or telephone calls. These changes in structure of the heart function clinics resulted in an initial drop in the total number of appointments, followed by a subsequent return to the usual visit volume to approximately 250 visits per month at UHN (97% virtual), 235 visits per month at MSH (35% virtual), and 115 visits per month at SHSC (34% virtual) by September 2020. Additionally, at UHN, patients were invited to enroll in the existing Medly Program, a mobile phone-based telemonitoring program designed to provide remote clinical support for patients with heart failure (HF).^5^ After 2 months of rapid patient onboarding in March and April 2020 (63 and 59 patients, respectively), the Medly Program had an average net growth of 30 patients per month from May to September 2020 (compared to 12 patients per month in the 12 months leading up to the COVID-19 pandemic). Telemonitoring was not available at MSH or SHSC.

### Study population

We screened adult patients (aged ≥ 18 years) who had presented to the ED with a triage diagnosis of “shortness of breath” and/or “leg swelling/edema,” and patients who were directly admitted to the hospital from an ambulatory clinic with an admitting diagnosis of ADHF, and including, of these patients, those who had ADHF. To qualify as having ADHF, all patients were required to have clinical records recording a diagnosis of ADHF based on clinical symptoms consistent with ADHF and either of the following: (i) a brain natriuretic peptide (BNP) level of ≥ 100 pg/mL or an N-terminal -proBNP (NT-proBNP) level of ≥ 300 pg/mL at the time of presentation; or (ii) a left ventricular ejection fraction (LVEF), as measured by echocardiography within the last year or during the index hospitalization, of ≤ 50%. Although the LVEF threshold of ≤ 50% was used as one inclusion criterion for identifying patients with ADHF, we did include patients with any LVEF if they satisfied one of the other inclusion criteria. Patients were excluded if they had an implantable ventricular assist device or a cardiac transplant, as they represent a unique cohort of patients for whom HF is managed by a specialized multidisciplinary team. Patients presenting with ADHF secondary to an acute coronary syndrome or secondary to severe pulmonary disease were also excluded. Informed consent was obtained for patients who presented after the study initiation (27 patients did not consent to participate and were thus excluded from the study sample); consent was waived for patients who presented to the hospital before study initiation. All study data were collected retrospectively.

### Data collection

We extracted clinical and laboratory data from electronic medical records, including the following: demographics (age, sex, residence location [forward sortation area of postal codes]; engagement with an HF program [ie, patient previously followed by an HF clinic]; full code status; comorbidities (ie, atrial fibrillation/flutter, diabetes mellitus, dyslipidemia, hypertension, smoking, chronic obstructive pulmonary disorder, liver cirrhosis, cancer, use of dialysis, dementia, peripheral vascular disease, cerebrovascular accident); HF characteristics and history (etiology of cardiomyopathy, time of initial HF diagnosis, pre-presentation New York Heart Association functional class, LVEF, HF medications at time of presentation, use of an implantable cardioverter defibrillator and/or cardiac resynchronization therapy); physical examination parameters (body mass index, heart rate, systolic blood pressure, respiratory rate); and laboratory data (hemoglobin, creatinine, sodium, and BNP/NT-proBNP levels). COVID-19 status was determined in the 2020 patient cohort according to institutional guidelines, based on nasopharyngeal swab testing and application of COVID-19 real-time reverse transcriptase-polymerase chain reaction.

### Outcomes

We collected information on the number of visits to the ED for patients with ADHF, along with admission rates for ADHF. Among patients admitted for ADHF, we evaluated the need for intensive care throughout the index admission, in-hospital all-cause mortality rates, and 30-day readmission or ED visit rate.

### Statistical analysis

Continuous variables are summarized by means and standard deviations, or medians and interquartile ranges (IQRs). Dichotomous and categorical variables are summarized as frequencies and proportions. We compared characteristics of patients seen during the 2020 pandemic to those of the 2019 time-matched cohort using Wilcoxon rank-sum tests or *t*-tests for continuous variables, and χ^2^ or Fisher’s exact tests for categorical variables. We used a generalized linear mixed model with random intercepts corrected for time as a weekly variable to estimate the average percent change in ED visits for ADHF, and hospitalizations for ADHF from the ED and from an HF clinic during the pandemic and previous year.

We used univariate and multivariate logistic regression models to evaluate whether the odds of hospitalization after presenting to the ED, 30-day readmission or ED visits, and in-hospital mortality differed by timeframe. We selected covariates based on their clinical importance and used automated forward (based on a *P* value < 0.15 on univariable analysis) and backward (based on a *P* value < 0.15 after entering multivariable analysis) for final variable selection to generate the multivariable regression models. We forced era, age, sex, and engagement with an HF program into the model. For in-hospital mortality, to account for patients with multiple admissions within a short timeframe, admissions that were within 7 days of the previous discharge date were considered to be one continuous admission, in which mortality occurring during the continuous hospitalization was considered to be in-hospital mortality. We also conducted simplified logistic regression models that included era and centre, to evaluate whether the probability of each of these outcomes in 2020 vs 2019 differed by centre. For outcomes in which a significant interaction was observed, we calculated pairwise estimates to determine the difference across eras at each centre for each outcome.

We considered a *P* value < 0.05 to be statistically significant. All statistical analyses were performed using Stata 16 (StataCorp, College Station, TX).

## Results

**Figure 1:**
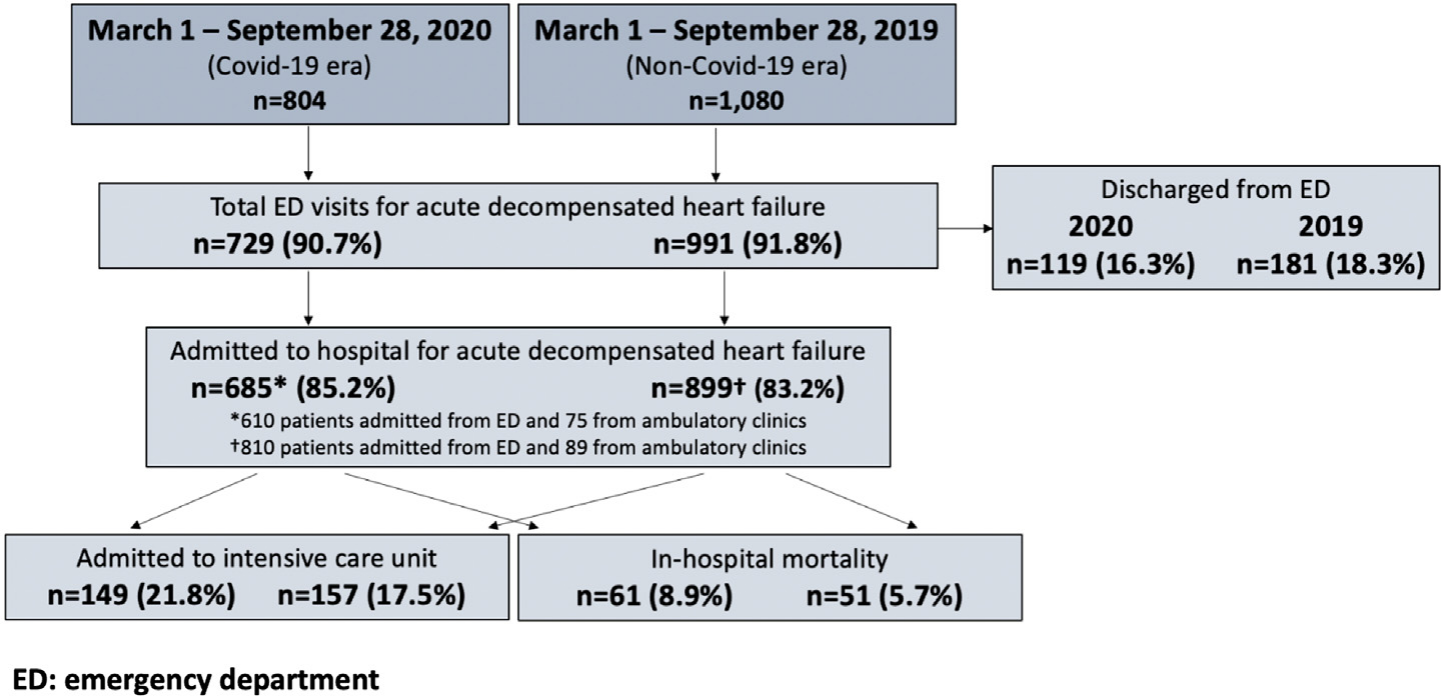
Patient flow through the emergency department (ED) into the hospital, and outcomes for patients with acute decompensated heart failure during the 2020 COVID-19 pandemic vs in 2019.

Overall, we recorded 1884 patients with ADHF who presented to the ED or were admitted directly from a clinic from March 1 to September 28, 2020, and the number of patients from the same timeframe in 2019. Of these patients, 804 presented during the 2020 COVID-19 pandemic, whereas 1080 presented during the 2019 study period (Fig. 1). The mean age of patients was 76 ± 15 years; 872 patients (46%) were female, and 391 patients (21%) were being followed by an HF program at the time of presentation to the hospital. Of the patients presenting in 2020, a total of 11 (1.4%) were identified as being positive for COVID-19. Supplemental Table S1 presents characteristics of these patients, overall and stratified by era. In general, patient characteristics were similar among those who presented to the hospital in 2020 vs in 2019.

### Characteristics of patients admitted to the hospital during the 2020 COVID-19 pandemic vs in 2019

Of the 1884 patients included in our study, 1584 patients were admitted to the hospital either from the ED or directly from a clinic, 685 during the 2020 COVID-19 pandemic, and 899 during the same timeframe in 2019. The median length of stay did not differ between patients admitted during the COVID-19 era vs those admitted in the previous year (7 days (IQR 4-13) vs 7 days (IQR 4-12), *P* = 0.27). Among this group of hospitalized patients (including those admitted from the ED and from a clinic in 2019 and 2020), the mean age was 76 ± 15 years; 748 patients (47%) were female, and 325 patients (21%) were being followed by an HF program at the time of admission. In general, patient characteristics were similar between the 2 eras, apart from the fact that more of the patients admitted in 2019 had a history of atrial fibrillation (Table 1). Of the patients admitted in 2020, a total of 10 (1.5%) were identified as being positive for COVID-19.

**Table 1:** Characteristics of patients admitted to the hospital with acute decompensated heart failure between March and September, in 2020 and 2019

Characteristic	Total (n = 1584)	COVID-19 era (n = 685)	Non-COVID-19 era (n = 899)	*P*
Age, y	76 ± 15	76 ± 14	76 ± 16	0.56
Female	748 (47.2)	316 (46.1)	432 (48.1)	0.48
Rural residency	24 (1.5)	12 (1.8)	12 (1.3)	0.54
Body mass index, kg/m^2^	27.7 ± 7.7	27.9 ± 7.6	27.7 ± 7.7	0.65
Race				0.17
Caucasian	734 (46.3)	316 (46.1)	418 (46.5)	
African-American	23 (1.5)	13 (1.9)	10 (1.1)	
Other	827 (52.2)	356 (52.0)	471 (52.4)	
Ischemic cardiomyopathy	475 (30.0)	210 (30.7)	265 (29.5)	0.62
Left ventricular ejection fraction, %	45 ± 17	45 ± 17	45 ± 17	0.49
< 40	588 (38.3)	247 (37.0)	341 (39.3)	0.56
40–60	670 (43.6)	292 (43.8)	378 (43.5)	
> 60	277 (18.0)	127 (19.1)	150 (17.3)	
NYHA functional class				0.16
I–II	1166 (74.0)	519 (75.9)	647 (72.6)	
III–IV	409 (26.0)	165 (24.1)	244 (27.4)	
Followed by a heart function program	325 (20.5)	139 (20.3)	186 (20.7)	0.85
Diagnosis of heart failure within 18 mo	850 (53.7)	383 (55.9)	467 (51.9)	0.13
Previous hospitalization for heart failure	811 (51.2)	347 (50.7)	464 (51.6)	0.72
COVID-19-positive	10 (0.6)	10 (1.5)	0 (0)	**0.001**
Coexisting conditions				
Atrial fibrillation	791 (49.9)	323 (47.2)	468 (52.1)	**0.05**
Diabetes	600 (37.9)	261 (38.1)	339 (37.7)	0.88
Dyslipidemia	787 (49.7)	352 (51.4)	435 (48.4)	0.24
Hypertension	1145 (72.3)	506 (73.9)	639 (71.1)	0.23
Active smoker	104 (6.6)	42 (6.1)	62 (6.9)	0.61
Chronic obstructive pulmonary disorder	263 (16.6)	101 (14.7)	162 (18.0)	0.09
Liver cirrhosis	49 (3.1)	25 (3.6)	24 (2.7)	0.31
Cancer	400 (25.3)	172 (25.1)	228 (25.4)	0.95
Use of dialysis	41 (2.6)	19 (2.8)	22 (2.4)	0.75
Dementia	134 (8.5)	55 (8.0)	79 (8.8)	0.65
Peripheral vascular disease	195 (12.3)	78 (11.4)	117 (13.0)	0.35
Cerebrovascular accident	232 (14.6)	110 (16.1)	122 (13.6)	0.17
Insulin use	302 (19.1)	138 (20.1)	164 (18.2)	0.65
QRS duration	119 ± 35	119 ± 36	118 ± 35	0.45
Full code status	925 (58.4)	394 (57.5)	531 (59.1)	0.54
Clinical measurements on initial presentation				
Systolic blood pressure at rest, mm Hg	130 ± 27	130 ± 27	129 ± 26	0.50
Heart rate at rest, bpm	86 ± 23	86 ± 23	87 ± 24	0.56
Respiratory rate, breaths/min	21 ± 6	22 ± 6	21 ± 6	0.12
Laboratory values				
Hemoglobin, g/L	116 ± 23	116 ± 24	116 ± 23	0.91
Sodium, mmol/L	138 ± 9	137 ± 11	138 ± 8	0.22
Serum creatinine, umol/L	119 (88–166)	122 (88–163)	117 (88–169)	0.47
Blood urea nitrogen, mmol/L	11 (8–18)	13 (7–19)	11 (8–17)	0.27
Brain natriuretic peptide, pg/mL	890 (370–1920)	780 (336–1873)	933 (437–1949)	0.11
NT-proB-type natriuretic peptide, pg/mL	3470 (1197–8800)	3488 (1510–9011)	3,470 (1025–8557)	0.18
Heart failure medication				
Beta-blockers	1044 (65.9)	455 (66.4)	589( 65.5)	0.71
ACEi, ARB, or ARNi	699 (44.1)	317 (46.3)	382 (42.5)	0.14
Spironolactone/eplerenone	318 (20.1)	140 (20.4)	178 (19.8)	0.75
Furosemide	968 (61.1)	422 (61.6)	546 (60.7)	0.75
Dose of furosemide, mg/d	60 (40–120)	60 (40–120)	60 (40–120)	0.85
Implantable cardioverter-defibrillator	164 (10.4)	68 (9.9)	96 (10.7)	0.68
Cardiac resynchronization therapy	62 (4.0)	31 (4.7)	31 (3.6)	0.30
Study centre				0.27
UHN	877 (55.4)	375 (54.8)	502 (55.8)	
MSH	145 (9.2)	55 (8.0)	90 (10.0)	
SHSC	562 (35.5)	254 (37.1)	308 (34.2)	

Values are expressed as n (%), mean ± standard deviation, or median (interquartile range), as appropriate, unless otherwise indicated.

ACEi, angiotensin-converting enzyme inhibitor; ARB:,angiotensin receptor blocker; ARNi, angiotensin receptor-neprilysin inhibitor; bpm: beats per minute; MSH, Mount Sinai Hospital; NT, N-terminal; NYHA, New York Heart Association; SHSC, Sunnybrook Health Sciences Centre; UHN, University Health Network.

### Absolute number of ED visits, hospital admissions, and outcomes during the 2020 COVID-19 pandemic vs in 2019

**Figure 2:**
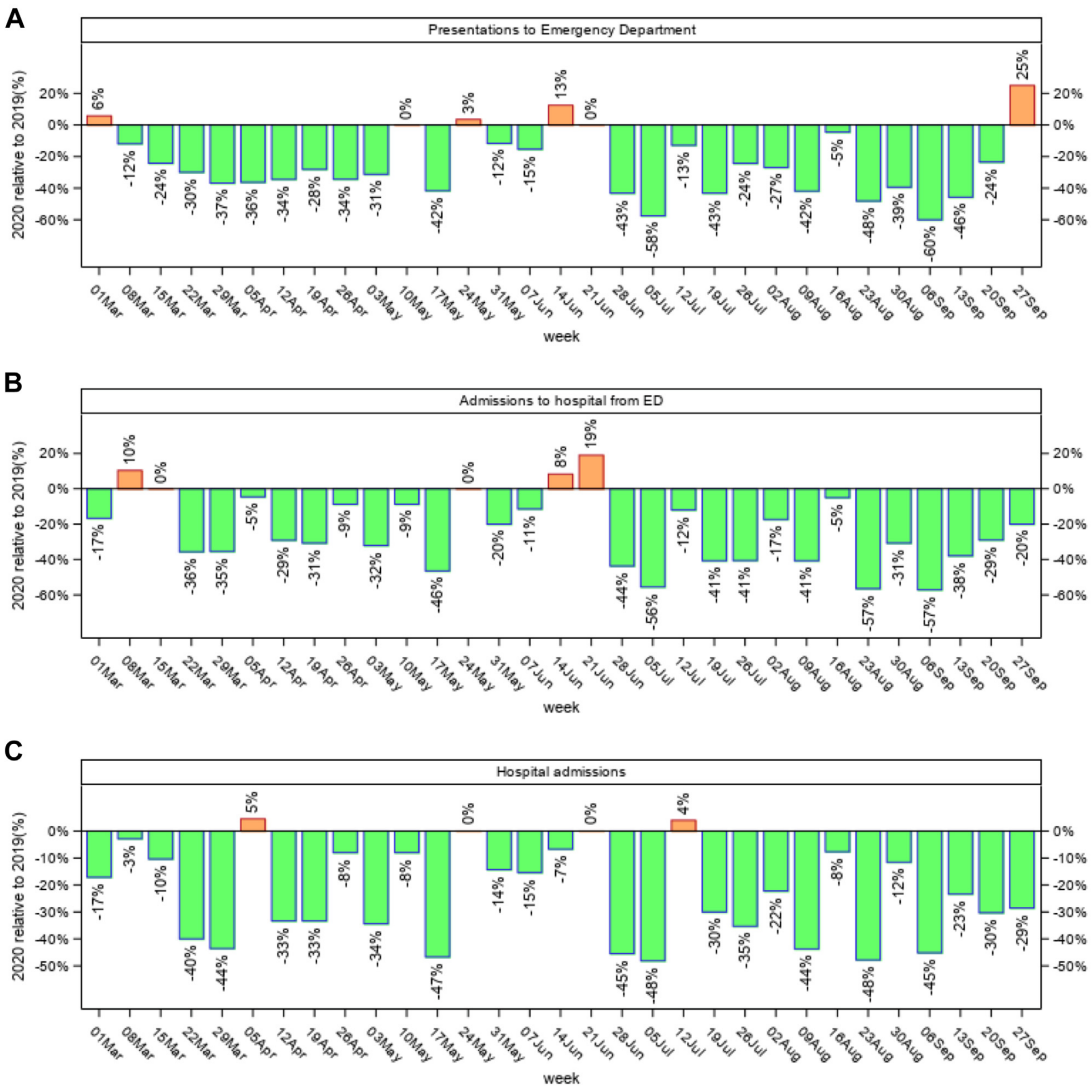
Average weekly decrease in the number of (**A**) presentations to the emergency department (ED), (**B**) admissions from the ED, and (**C**) all admissions (from ED and directly from a clinic) between 2019 and 2020.

Between March 1 and September 28, 2020, we recorded a numerically lower total number of ED visits for ADHF, compared with the number of ED visits from the same timeframe in 2019 (729 vs 991, respectively; Table 2). Among patients presenting to the ED, the absolute number of admissions from the ED were lower in 2020 compared to that in 2019 (610 and 810, respectively), as was the absolute number of patients admitted directly from a clinic—75 patients in 2020 vs 89 in 2019. Compared to 2019, the average weekly number of ED visits in 2020 decreased by 26% (95% confidence interval [CI] 19% to 32%), the average weekly number of admissions from an ED visit decreased by 25% (95% CI 16% to 32%), and the average weekly number of all admissions (from an ED and directly from a clinic) decreased by 18% (95% CI 12% to 40%; Fig. 2).

**Table 2:** Number of patients who presented to the emergency department (ED), were admitted, were readmitted, or died, in 2020 vs 2019

Among all visits	Total (n = 1884)	COVID-19 era (n = 804)	Non-COVID-19 era (n = 1080)	*P*
Presented to ED	1720 (91.3)	729 (90.7)	991 (91.8)	0.41
Were admitted to hospital	1584 (84.1)	685 (85.2)	899 (83.2)	0.28
Among all admissions only				
Median length of stay, d	7 (4–13)	7 (4–13)	7 (4–12)	0.27
Admitted to ICU	306 (19.3)	149 (21.8)	157 (17.5)	0.03
In-hospital mortality	112 (7.1)	61 (8.9)	51 (5.7)	0.01
Repeat admission within 30 days of discharge	98 (6.6)	43 (6.9)	55 (6.5)	0.75
Repeat admission or ED visit within 30 days of discharge	112 (7.6)	49 (7.8)	63 (7.4)	0.77

Values are n (%) or median (interquartile range), unless otherwise indicated.

ICU, intensive care unit.

### Probability of admission after ED presentation and outcomes during hospitalization in the 2020 COVID-19 pandemic vs in 2019

Following a visit to the ED, the probability of being admitted to the hospital did not differ for patients seen in 2020 vs in 2019. Multivariate analyses indicate that the odds of being admitted to the hospital following presentation to the ED were not significantly increased (odds ratio (OR) 1.15, 95% CI 0.88 to 1.49) in 2020 vs the odds in 2019 after adjusting for age, sex, residence location, engagement with an HF program, LVEF, New York Heart Association class, history of dyslipidemia, history of dementia, history of peripheral vascular disease, systolic blood pressure, heart rate, respiratory rate, and hemoglobin and creatinine levels (Tables 3 and 4). In a simplified model including era and centre evaluating the probability of admission after ED presentation (Supplemental Table S2), we identified a significant interaction effect, suggesting that the probability in 2020 vs 2019 differs between UHN and SHSC (OR 1.50, 95% CI 1.08 to 2.1 at UHN; OR 0.83, 95% CI 0.36 to 1.88 at MSH; OR 0.66, 95% CI 0.41 to 1.06 at SHSC, for interaction effect *P* < 0.02).

**Table 3:** Three logistic regression models evaluating the impact of COVID-19 on the probability of admission to the hospital among all presentations to the emergency department (ED), in-hospital mortality among all admissions to the hospital, and 30-day readmission or ED visit among all admissions to the hospital of patients discharged alive, evaluated by comparing patients presenting in 2020 vs in 2019

Outcome	Unadjusted OR (95% CI)	*P*	Adjusted∗ OR (95% CI)	*P*
Admission to the hospital, among all presentations to the ED	1.145 (0.888–1.477)	0.2948	1.147 (0.882–1.493)	0.3061
In-hospital mortality, among all admissions to the hospital	1.649 (1.113–2.443)	0.0126	1.657 (1.083–2.533)	0.0198
30-day readmission or ED visit, among all admissions to the hospital of patients who were discharged alive	1.064 (0.722–1.569)	0.7541	1.078 (0.729–1.595)	0.7072

CI, confidence interval; OR, odds ratio.

∗ Multivariate model was adjusted for multiple baseline characteristics, comorbidities, laboratory values, and heart failure medications as described in Table 4 and Supplemental Tables S3 and S4.

**Table 4:** Univariable and multivariable logistic regression results for admission to the hospital, among all presentations to the emergency department

Variable	Univariable OR (95% CI)	*P*	Multivariable OR (95% CI)	*P*
2020 (ref: 2019)	1.145 (0.888–1.477)	0.2948	1.147 (0.882–1.493)	0.3061
Age, per 1-y increase	1.017 (1.009–1.025)	< 0.0001	1.012 (1.003–1.021)	0.0091
Female (ref: male)	1.342 (1.042–1.726)	0.0224	1.227 (0.927–1.625)	0.1521
Rural residency (ref: not rural)	3.401 (0.450–25.704)	0.2355	5.242 (0.677–40.579)	0.1126
Engagement with HF program	0.798 (0.589–1.082)	0.1471	0.892 (0.643–1.237)	0.4925
Body mass index, per kg/m^2^ increase	1.007 (0.991–1.023)	0.3990	—	—
Ischemic cardiomyopathy	0.981 (0.748–1.286)	0.8894	—	—
Left ventricular ejection fraction, per % increase	1.013 (1.006–1.021)	0.0006	1.011 (1.002–1.02)	0.0184
NYHA functional class III or IV (ref: I or II)	0.778 (0.588–1.029)	0.0781	0.751 (0.557–1.013)	0.0605
Recent diagnosis of HF within 18 mo	1.201 (0.936–1.542)	0.1494	—	—
COVID-19 positive	1.907 (0.241–15.11)	0.5410	—	—
Coexisting conditions	—	—	—	—
Atrial fibrillation/flutter	0.973 (0.758–1.248)	0.8292	—	—
Diabetes	1.316 (1.009–1.717)	0.0428	—	—
Dyslipidemia	1.351 (1.050–1.737)	0.0192	1.339 (1.027–1.746)	0.0312
Hypertension	1.489 (1.141–1.944)	0.0034	—	—
Active smoking (ref: former or never)	0.780 (0.492–1.235)	0.2888	—	—
Chronic obstructive pulmonary disease	1.128 (0.803–1.585)	0.4880	—	—
Liver cirrhosis	1.057 (0.511–2.186)	0.8808	—	—
Cancer	1.168 (0.868–1.572)	0.3056	—	—
Use of dialysis	1.446 (0.561–3.729)	0.4449	—	—
Dementia	1.683 (0.984–2.878)	0.0571	1.546 (0.888–2.693)	0.1238
Peripheral vascular disease	2.294 (1.371–3.839)	0.0016	2.328 (1.372–3.950)	0.0017
Cerebrovascular accident	0.978 (0.690–1.387)	0.9001	—	—
Full code status	0.771 (0.597–0.996)	0.0467	—	—
Clinical measurements on initial presentation	—	—	—	—
Systolic blood pressure, per mm Hg increase	0.999 (0.994–1.003)	0.6046	0.994 (0.988–0.999)	0.0151
Heart rate, per bpm increase	1.009 (1.003–1.015)	0.0033	1.013 (1.006–1.019)	0.0002
Respiratory rate, per bpm increase	1.058 (1.028–1.088)	0.0001	1.042 (1.013–1.073)	0.0047
QRS duration, per ms increase	0.998 (0.995–1.002)	0.3284	—	—
Laboratory values	—	—	—	—
Hemoglobin, per g/L increase	0.990 (0.985–0.996)	0.0006	0.995 (0.989–1.001)	0.1197
Sodium, per meq/L increase	0.990 (0.975–1.004)	0.1657	—	—
Creatinine, per μmol/dL increase	1.002 (1.001–1.004)	0.0051	1.002 (1.000–1.004)	0.0103
HF medication	—	—	—	—
Beta-blocker	0.801 (0.611–1.05)	0.1074	—	—
ACEi, ARB, or ARNi	0.782 (0.609–1.003)	0.0529	—	—
Mineralocorticoid receptor antagonist	0.572 (0.427–0.766)	0.0002	—	—
Implantable cardioverter defibrillator	0.577 (0.397–0.84)	0.0041	—	—
Cardiac resynchronization therapy	0.801 (0.407–1.574)	0.5192	—	—
Dose of furosemide, mg/d	1.000 (0.998–1.002)	0.8337	—	—

ACEi, angiotensin-converting enzyme inhibitors; ARB, angiotensin receptor blocker; ARNi, angiotensin receptor-neprilysin inhibitor; bpm, beats per minute; CI, confidence interval; ED, emergency department; HF, heart failure; NYHA, New York Heart Association; OR, odds ratio; ref, referent.

Among ADHF patients admitted to the hospital, we observed a significantly higher proportion of patients being admitted to the intensive care unit (ICU) during the 2020 COVID-19 pandemic vs in 2019 (21.8% vs 17.5%, *P* = 0.03). In-hospital mortality was significantly higher among the patients admitted during the COVID-19 era vs in 2019 (8.9% vs 5.7%, *P* = 0.01). Multivariable analysis indicates that the odds of in-hospital mortality was 66% higher (OR 1.66, 95% CI 1.08 to 2.53) among patients who were admitted to the hospital in 2020 vs in 2019 (Table 3; Supplemental Table S3). No differences in in-hospital mortality by era were found across centres (Supplemental Table S2).

Multivariable analysis indicates that after hospital discharge, the odds of 30-day readmission or recurrent ED visit among patients discharged alive was not significantly increased (OR 1.08, 95% CI 0.73 to 1.60) in 2020 vs 2019 (Table 3; Supplemental Table S4).

### Outcomes for patients presenting to the hospital with ADHF and COVID-19-positive status

We identified 11 patients (1.4%) who presented to the hospital with ADHF during the 2020 COVID-19 pandemic who had a positive COVID-19 status. Of these patients, 10 presented to the ED, and 1 patient was admitted directly from a clinic. All patients were admitted to the hospital, except for 1 patient who left against medical advice and was alive at 30 days post-ED visit. Among the patients admitted to the hospital, 1 patient was admitted to the ICU (and discharged alive), and 1 patient died in-hospital from COVID-19-related pneumonia.

## Discussion

### Summary of findings

Previously, we reported a decline in ADHF-related ED visits and hospitalizations during the early stages of the 2020 COVID-19 pandemic, vs the number in 2019.^4^ In the current study, which includes 2 additional study centres, as well as a longer timeframe, we continued to observe a lower total number of patients presenting with ADHF to the ED at 3 tertiary care centres in Toronto, Canada, throughout the COVID-19 pandemic, as compared to a time-matched cohort in 2019. Despite the lower volume of patients presenting to the ED and the lower absolute number of admissions to the hospital for ADHF, the probability of being admitted to the hospital did not differ for patients seen in 2020 vs in 2019 after adjusting for several important covariates. Among ADHF patients admitted to the hospital, however, we observed a significantly higher proportion of patients being admitted to the ICU, as well as a relative 66% increase and an absolute 3% increase in in-hospital mortality during the 2020 COVID-19 era vs in 2019.

### Relation to previous work

Throughout the COVID-19 pandemic, healthcare centres around the world have had to restructure their delivery of HF care in unprecedented ways to effectively manage patients while minimizing the risk of COVID-19 transmission. In efforts to reduce the spread of the novel severe acute respiratory syndrome coronavirus-2 (SARS-CoV-2) infection, HF centres have transitioned to virtual care platforms, and in some cases, deferred or delayed patient management.^1^ Although the consequences of the SARS-CoV-2 infection are profound, the indirect effects of the COVID-19 pandemic, including delay or foregoing of health care for patients living with other chronic conditions, are believed to carry an equal, if not greater burden on subsequent cardiovascular mortality and morbidity.^1^^,^^6^

Our study findings demonstrate that ADHF presentations to healthcare facilities have decreased during the COVID-19 pandemic, and as a result, these patients may be increasing their risk of further HF progression and mortality. These observations parallel our findings from the early stages of the COVID-19 pandemic^4^ and demonstrate that the trend in reductions of both ADHF presentations and admissions to the hospital has continued throughout this 7-month study period. Many other studies have reported reductions in ADHF-related ED visits and hospital admissions since the start of the COVID-19 pandemic,^7^, ^8^, ^9^, ^10^ and this phenomenon has been observed across other non-COVID-19-related conditions also,^3^^,^^11^, ^12^, ^13^ including acute cardiovascular conditions such as stroke,^14^^,^^15^ and myocardial infarction.^16^^,^^17^ Although we are not able to confirm the reasons for the observed behaviour in this study, it is likely a consequence of the collective pandemic response and the fear imposed by public health messaging regarding stay-at-home orders.^3^^,^^6^

Among the patients who did present to the hospital in our study, the probability of admission (ie, receiving in-hospital care for HF management) was no different between 2019 and 2020. Moreover, we did not observe any difference in the risk of readmission or recurrent ED visits within 30 days of discharge during the COVID-19 pandemic vs in 2019. This finding may suggest that the resources available to patients and the quality of HF care have not changed, despite the shift in healthcare practices during the COVID-19 pandemic. This notion should be emphasized in public health messaging, as patients living with chronic health conditions, including HF, should be encouraged to seek timely medical care when needed throughout the pandemic. Given the increased rates of ICU admission and in-hospital mortality observed among patients who were hospitalized for ADHF in 2020, compared to those in 2019, our study demonstrates that patients are likely waiting too long at home before presenting to the hospital. This theory is further supported by the fact that ICU resources were not exhausted, and no additional triage strategies were implemented, across the 3 study institutions during this time. Only 1 death in our study was attributed to COVID-19, which makes us wonder if many of the other deaths we observed could have been avoidable given stronger public health messaging and earlier presentation to the hospital.^13^

Other centres around the world have demonstrated similar increases in in-hospital mortality rates since the start of the COVID-19 pandemic. We observed a 66% increase in the odds of in-hospital mortality among patients admitted to the hospital during the COVID-19 era vs in 2019. Similarly, Germany has reported a 27% increase in the relative risk of in-hospital mortality for March-April 2020 vs 2 control periods,^6^ and another study from the United Kingdom demonstrated a 2-fold increase in in-hospital mortality from January-June 2020, compared to that in the same timeframe in 2019.^8^ In Canada, similar trends have been reported by the Canadian Cardiovascular Society COVID-19 Challenge for Canada Initiative (CCS-C3I), which has demonstrated an ∼30% decrease in HF admissions, and increased in-hospital mortality rates from 9.5% (April 2019) to 10.5% (April 2020), for patients with HF in Canada during this first wave of the COVID-19 pandemic.^18^

### Clinical implications

Altogether, these findings have important implications for the clinical care of patients living with HF and other chronic conditions. Given the significantly worse patient outcomes observed during the COVID-19 era compared to those in previous time-matched control periods, critical to the approach taken in the event of other emergent situations, be they COVID-19-related or not, is the development and adoption of stronger patient-management strategies. In Ontario, approximately 21,000 adults are admitted to the hospital with HF each year,^19^ and of these patients, approximately 11% will die in-hospital (2300 patients per year).^20^ Applying the findings on increased in-hospital mortality risk from these 3 institutions to other institutions across Ontario, the results from our study suggest that the changes in patient care seen as a result of the public measures and the subsequent fear developed during this pandemic may have led to approximately 1500 additional HF-related deaths in Ontario in 2020. This simple mathematical impact analysis does not consider the reduction in the number of hospital admissions and the possible unmeasurable increase in HF-related deaths occurring outside the hospital setting during the COVID-19 era (ie, HF patients dying at home). Although these numbers are specific to the HF population, similar suboptimal care of patients living with other diseases also has been reported during the COVID-19 pandemic.^14^, ^15^, ^16^, ^17^

### Limitations

Our study has limitations. The COVID-19 pandemic has affected individual centres differently. This heterogeneity, as demonstrated by the interaction effect we observed regarding the probability of admission after ED presentation across the 3 study centres, may increase the chances of our study results being representative of other academic centres in Ontario. Our results, however, may not be generalizable to nonacademic medical centres. The number of patients presenting to the ED could have been underestimated, as patients who presented with triage diagnosis codes other than “shortness of breath” or “leg swelling/edema” were not screened for inclusion in this study. However, such underestimation would have affected both the 2020 and 2019 study cohorts, thereby imposing minimal bias on the study findings. An underestimation of the number of events reported is also possible, namely readmissions and deaths, as some patients might have presented to a different hospital for their follow-up care, or died at home. Moreover, patients who did not present to the hospital at all could not be captured in this study, possibly leading to an underestimation of ADHF-related mortality during the COVID-19 pandemic if these patients died outside of the hospital setting. Another possibility, which may even be likely, is that patients who did not seek medical attention for their ADHF were at an increased risk of mortality, compared with those who presented to the hospital during the COVID-19 pandemic. We also did not have access to disease-specific outcome data.

The findings presented in this article also may be influenced by the virtual care programs established at each of these 3 institutions. Although we were not able to explore this possibility in the current study, the observed reductions in ED visits and admissions during the COVID-19 pandemic could have been influenced by an increase in virtual appointments since the start of the pandemic. Also possible is that the virtual care system did not allow for adequate recognition of patients at higher risk, thereby contributing to the increased in-hospital mortality rates observed in this study. Perhaps HF patients with a higher comorbidity burden and more pronounced cardiac dysfunction should, at a minimum, be required to have a scheduled face-to-face interaction periodically, as opposed to treatment provided primarily via a virtual care system. A recent study from the US, however, has demonstrated that the telehealth model for managing outpatients with HF during the COVID-19 pandemic was safe and effective and did not result in increases in acute HF care or mortality.^21^

## Conclusions

During the COVID-19 pandemic, we observed a lower volume of patients presenting to the ED with ADHF, and a lower absolute number of ADHF admissions, as compared to those in a time-matched cohort in 2019. Despite these trends, the probability of being admitted to the hospital did not differ, but the risk of in-hospital mortality was significantly higher during the 2020 COVID-19 era vs in 2019. Our findings suggest that improved messaging may be needed for patients living with chronic health conditions, including HF, during the pandemic, to educate and encourage them to present to hospital services when in need.
