# Supplementary material for: Increased Mortality in Patients With Acutely Decompensated Heart Failure During the COVID-19 Pandemic in Toronto, Canada
Source: CJC Open. 2022 Jun 22;4(9):772–81. doi: 10.1016/j.cjco.2022.06.006 (PMC9221741; doi:10.1016/j.cjco.2022.06.006)
Supplement: Supplementary Material [file mmc1.docx]

**SUPPLEMENTAL MATERIAL**

**Supplemental Figure S1: Number of Covid-19 cases in Ontario and main public health measures**

**
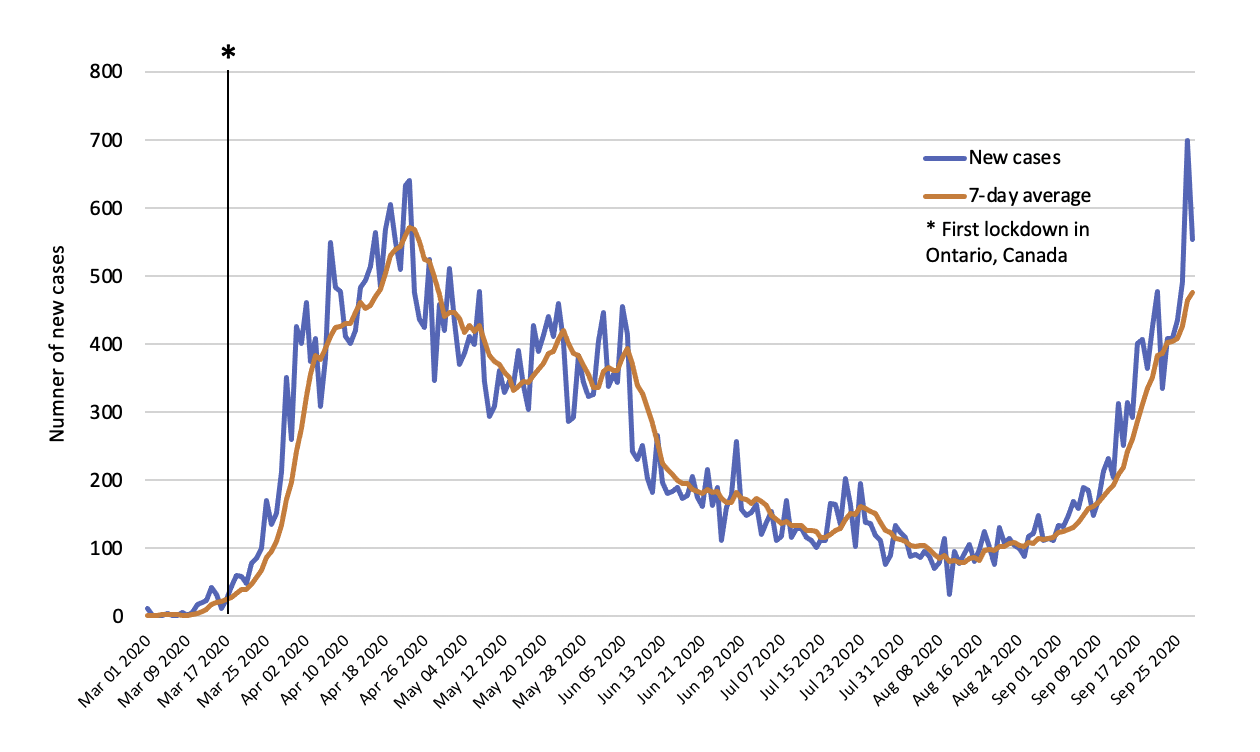
**

**Supplemental Table S1: Characteristics of patients presenting to hospital (to the ED or admitted directly from clinic) with acute decompensated heart failure between March to September in 2020 and 2019**

| **Characteristic** | **Total**  **(n=1,884)** | **Covid-19 era (n=804)** | **non-Covid-19 era (n=1,080)** | **p-value** |
| --- | --- | --- | --- | --- |
| Age (years) | 76 ± 15 | 76 ± 14 | 75 ± 16 | 0.17 |
| Female | 872 (46.3%) | 365 (45.4%) | 507 (46.9%) | 0.51 |
| Rural residency | 24 (1.3%) | 12 (1.5%) | 12 (1.1%) | 0.54 |
| Body mass index (kg/m2) | 27.8 ± 7.6 | 27.8 ± 7.5 | 27.7 ± 7.8 | 0.91 |
| Race |  |  |  | 0.26 |
| Caucasian | 872 (46.3%) | 374 (46.5%) | 498 (46.1%) |  |
| African-American | 29 (1.5%) | 16 (2.0%) | 13 (1.2%) |  |
| Other | 983 (52.2%) | 414 (51.5%) | 569 (52.7%) |  |
| Ischemic Cardiomyopathy | 566 (30.0%) | 262 (32.6%) | 304 (28.1%) | 0.04 |
| Left Ventricular Ejection Fraction (%) | 44 ± 17 | 45 ± 17 | 44 ± 17 | 0.38 |
| <40% | 709 (39.2%) | 289 (37.5%) | 420 (40.5%) | 0.44 |
| 40-60% | 780 (43.1%) | 343 (44.5%) | 437 (42.1%) |  |
| >60% | 319 (17.6%) | 138 (17.9%) | 181 (17.4%) |  |
| NYHA functional class |  |  |  | 0.004 |
| I - II | 1,380 (73.7%) | 605 (75.3%) | 775 (72.4%) |  |
| III - IV | 493 (26.3%) | 198 (24.7%) | 295 (27.6%) |  |
| Followed by a Heart Function Program | 391 (20.8%) | 165 (20.5%) | 226 (20.9%) | 0.86 |
| Diagnosis of heart failure within 18 months | 1,003 (53.2%) | 445 (55.3%) | 558 (51.7%) | 0.12 |
| Previous hospitalization for heart failure | 979 (52.0%) | 409 (50.9%) | 570 (52.8%) | 0.43 |
| COVID-19 positive | 11 (0.6%) | 11 (1.4%) | 0 (0%) | <0.001 |
| **Coexisting conditions** |  |  |  |  |
| Atrial fibrillation | 942 (50.0%) | 384 (47.8%) | 558 (51.7%) | 0.10 |
| Diabetes | 695 (36.9%) | 302 (37.6%) | 393 (36.4%) | 0.63 |
| Dyslipidemia | 914 (48.5%) | 402 (50.0%) | 512 (47.4%) | 0.28 |
| Hypertension | 1,342 (71.2%) | 592 (73.6%) | 750 (69.4%) | 0.05 |
| Active smoker | 129 (6.8%) | 48 (6.0%) | 81 (7.5%) | 0.20 |
| Chronic Obstructive Pulmonary Disorder | 310 (16.5%) | 115 (14.3%) | 195 (18.1%) | 0.03 |
| Liver Cirrhosis | 58 (3.1%) | 30 (3.7%) | 28 (2.6%) | 0.18 |
| Cancer | 467 (24.8%) | 197 (24.5%) | 270 (25.0%) | 0.83 |
| Use of Dialysis | 46 (2.4%) | 21 (2.6%) | 25 (2.3%) | 0.76 |
| Dementia | 150 (8.0%) | 61 (7.6%) | 89 (8.2%) | 0.67 |
| Peripheral Vascular Disease | 212 (11.3%) | 84 (10.4%) | 128 (11.9%) | 0.38 |
| Cerebrovascular Accident | 277 (14.7%) | 132 (16.4%) | 145 (13.4%) | 0.08 |
| Insulin use | 341 (40.2%) | 154 (41.2%) | 187 (39.5%) | 0.62 |
| QRS Duration | 119 ± 36 | 120 ± 36 | 118 ± 35 | 0.15 |
| Full Code Status | 1,113 (59.1%) | 469 (58.3%) | 644 (59.6%) | 0.60 |
| **Clinical measurements on initial presentation** |  |  |  |  |
| Systolic Blood Pressure at Rest (mmHg) | 130 ± 27 | 131 ± 27 | 130 ± 26 | 0.41 |
| Heart Rate at Rest (bpm) | 86 ± 23 | 85 ± 22 | 86 ± 23 | 0.18 |
| Respiratory Rate (breaths/min) | 21 ± 6 | 21 ± 6 | 21 ± 7 | 0.23 |
| **Laboratory values** |  |  |  |  |
| Hemoglobin (g/L) | 116 ± 23 | 117 ± 24 | 116 ± 22 | 0.74 |
| Sodium (mmol/L) | 138 ± 9 | 137 ± 10 | 138 ± 7 | 0.25 |
| Serum Creatinine (umol/L) | 115 (86 – 160) | 119 (88 – 160) | 113 (84 – 160) | 0.20 |
| Brain Natriuretic Peptide (pg/mL) | 872 (370 – 1,880) | 728 (323 – 1,755) | 933 (437 – 2,034) | 0.02 |
| NT-proB-type Natriuretic Peptide (pg/mL) | 3,241 (1,130 – 8,403) | 3,360 (1,270 – 8,197) | 3,065 (912 – 8,479) | 0.14 |
| **Heart failure medication** |  |  |  |  |
| B-Blockers | 1,255 (66.6%) | 540 (67.2%) | 715 (66.2%) | 0.69 |
| ACEi, ARB, or ARNi | 849 (45.1%) | 381 (47.4%) | 468 (43.3%) | 0.08 |
| Spironolactone/Eplerenone | 397 (21.1%) | 176 (21.9%) | 221 (20.5%) | 0.46 |
| Furosemide | 1,171 (62.2%) | 504 (62.7%) | 667 (61.8%) | 0.70 |
| Dose of Furosemide | 60 (40 – 100) | 60 (40 – 90) | 60 (40 – 120) | 0.69 |
| Implantable Cardioverter-Defibrillator | 206 (10.9%) | 85 (10.6%) | 121 (11.2%) | 0.71 |
| Cardiac Resynchronization Therapy | 73 (4.0%) | 37 (4.7%) | 36 (3.4%) | 0.18 |
| **Study centre** |  |  |  | 0.05 |
| UHN | 1,069 (56.7%) | 437 (54.4%) | 632 (58.5%) |  |
| MSH | 174 (9.2%) | 68 (8.5%) | 106 (9.8%) |  |
| SHSC | 641 (34.0%) | 298 (37.1%) | 343 (31.7%) |  |

Values expressed as n (%), mean ± SD, and median (IQR) as appropriate.

ACEi: angiotensin converting enzyme inhibitors; ARB: angiotensin receptor blockers; ARNi: angiotensin receptor-neprilysin inhibitor; bpm: beats per minute; ED: emergency department; MSH: Mount Sinai Hospital; SHSC: Sunnybrook Health Sciences Centre; UHN: University Health Network.

**Supplemental Table S2: Three logistic regression models evaluating the interaction between era and center to evaluate the probability of (A) admission after ED presentation, (B) in-hospital mortality, and (C) 30-day readmission or ED visit in 2020 versus 2019 across the 3 centers**

**A. Admission to hospital, among all presentations to the ED**

| **Factor** | **Univariable OR (95% CI)** | **P-value** |
| --- | --- | --- |
| **Center by era (2020 vs. 2019)** |  | **0.0162** |
| **UHN** | 1.502 (1.075 – 2.100) | **0.002** |
| **MSH** | 0.826 (0.364 – 1.876) | 0.18 |
| **SHSC** | 0.659 (0.410 – 1.061) | 0.16 |

**B. In-hospital mortality, among all admissions to hospital**

| **Factor** | **Univariable OR (95% CI)** | **P-value** |
| --- | --- | --- |
| **Center by era (2020 vs. 2019)** |  | 0.33 |
| **UHN** | 1.100 (0.569 – 2.130) |  |
| **MSH** | 2.004 (1.200 – 3.347) |  |
| **SHSC** | 2.510 (0.406 – 15.518) |  |

**C. 30-day readmission or ED visit, among all admissions to hospital who were discharged alive**

| **Factor** | **Univariable OR (95% CI)** | **P-value** |
| --- | --- | --- |
| **Center by era (2020 vs. 2019)** |  | 0.77 |
| **UHN** | 1.243 (0.670 – 2.306) |  |
| **MSH** | 0.921 (0.534 – 1.589) |  |
| **SHSC** | 1.139 (0.306 – 4.239) |  |

**Supplemental Table S3: Univariable and multivariable logistic regression results for in-hospital mortality, among all admissions to hospital**

| **Variable** | **Univariable**  **OR (95% CI)** | **p-value** | **Multivariable**  **OR (95% CI)** | **p-value** |
| --- | --- | --- | --- | --- |
| 2020 (ref: 2019) | 1.649 (1.113 - 2.443) | 0.0126 | 1.657 (1.083 - 2.533) | 0.0198 |
| Age (per 1-year increase) | 1.026 (1.010 - 1.042) | 0.0012 | 1.014 (0.996 - 1.033) | 0.1172 |
| Female (ref: Male) | 1.606 (1.081 - 2.387) | 0.0191 | 1.497 (0.965 - 2.324) | 0.0718 |
| Rural residency (ref: not Rural) | 1.935 (0.568 - 6.592) | 0.2913 | -- | -- |
| Engagement with HF program | 0.715 (0.420 - 1.219) | 0.2176 | 0.643 (0.356 - 1.162) | 0.1436 |
| Body mass index (per kg/m^2^ increase) | 0.995 (0.970 - 1.02) | 0.6821 | -- | -- |
| Ischemic cardiomyopathy | 1.566 (1.046 - 2.343) | 0.0293 | -- | -- |
| Left ventricular ejection fraction (per % increase) | 0.997 (0.986 - 1.009) | 0.6527 | -- | -- |
| NYHA functional class III or IV (ref: I or II) | 2.173 (1.455 - 3.244) | 0.0001 | 2.229 (1.424 - 3.489) | 0.0005 |
| Recent diagnosis of heart failure within 18 months | 0.671 (0.452 - 0.994) | 0.0465 | -- | -- |
| Covid-19 positive | 1.489 (0.187 - 11.864) | 0.7069 | -- | -- |
| **Coexisting conditions** | -- | -- | -- | -- |
| Atrial Fibrillation/Flutter | 0.861 (0.582 - 1.274) | 0.4539 | 0.654 (0.422 - 1.013) | 0.0569 |
| Diabetes | 1.126 (0.756 - 1.677) | 0.5588 | -- | -- |
| Dyslipidemia | 0.962 (0.651 - 1.423) | 0.8473 | -- | -- |
| Hypertension | 1.111 (0.712 - 1.734) | 0.6434 | -- | -- |
| Active smoking (ref: former or never) | 0.259 (0.063 - 1.065) | 0.0611 | -- | -- |
| Chronic obstructive pulmonary disease | 0.946 (0.553 - 1.617) | 0.8387 | -- | -- |
| Liver Cirrhosis | 1.223 (0.431 - 3.47) | 0.7051 | -- | -- |
| Cancer | 1.267 (0.824 - 1.947) | 0.281 | -- | -- |
| Use of Dialysis | 1.462 (0.511 - 4.179) | 0.479 | -- | -- |
| Dementia | 1.688 (0.933 - 3.052) | 0.0833 | -- | -- |
| Peripheral Vascular Disease | 0.716 (0.367 - 1.399) | 0.3285 | 0.563 (0.268 - 1.181) | 0.1288 |
| Cerebrovascular Accident | 2.227 (1.412 - 3.513) | 0.0006 | 2.08 (1.257 - 3.442) | 0.0044 |
| Full code status | 0.201 (0.128 - 0.317) | <.0001 | 0.212 (0.129 - 0.348) | <0.0001 |
| **Clinical measurements on initial presentation** | -- | -- | -- | -- |
| Systolic blood pressure (per mmHg increase) | 0.981 (0.973 - 0.989) | <.0001 | 0.975 (0.966 - 0.984) | <0.0001 |
| Heart rate (per bpm increase) | 1.003 (0.995 - 1.011) | 0.5145 | -- | -- |
| Respiratory rate (per bpm increase) | 1.029 (1.003 - 1.056) | 0.0286 | -- | -- |
| QRS duration (per ms increase) | 1.001 (0.995 - 1.006) | 0.8131 | -- | -- |
| **Laboratory values** | -- | -- | -- | -- |
| Hemoglobin (per g/L increase) | 0.993 (0.985 - 1.002) | 0.1233 | -- | -- |
| Sodium (per meq/L increase) | 0.991 (0.972 - 1.011) | 0.3844 | -- | -- |
| Creatinine (per µmol/dL increase) | 1.002 (1.001 - 1.003) | <.0001 | 1.003 (1.002 - 1.005) | <0.0001 |
| **Heart failure medication** | -- | -- | -- | -- |
| Beta-blocker | 0.743 (0.499 - 1.108) | 0.1449 | -- | -- |
| ACEi, ARB, or ARNi | 0.694 (0.462 - 1.041) | 0.0773 | -- | -- |
| Mineralocorticoid receptor antagonist | 0.530 (0.293 - 0.960) | 0.0362 | 0.553 (0.288 - 1.061) | 0.0749 |
| Implantable cardioverter defibrillator | 0.580 (0.265 - 1.271) | 0.1734 | -- | -- |
| Cardiac resynchronization therapy | 1.202 (0.471 - 3.067) | 0.6996 | -- | -- |
| Dose of Furosemide | 1.001 (0.998 - 1.004) | 0.6367 | -- | -- |

ACEi: angiotensin converting enzyme inhibitors; ARB: angiotensin receptor blockers; ARNi: angiotensin receptor-neprilysin inhibitor; CI: confidence interval; ED: emergency department; OR: odds ratio

**Supplemental Table S4: Univariable and multivariable logistic regression results for 30-day readmission or ED visit, among all admissions to hospital whether from ED or not that resulted in discharges alive**

| **Variable** | **Univariable**  **OR (95% CI)** | **p-value** | **Multivariable**  **OR (95% CI)** | **p-value** |
| --- | --- | --- | --- | --- |
| 2020 (ref: 2019) | 1.064 (0.722 - 1.569) | 0.7541 | 1.078 (0.729 - 1.595) | 0.7072 |
| Age (per 1-year increase) | 0.998 (0.986 - 1.011) | 0.765 | 1.000 (0.987 - 1.014) | 0.9569 |
| Female (ref: Male) | 0.791 (0.535 - 1.169) | 0.2396 | 0.891 (0.595 - 1.335) | 0.5761 |
| Rural residency (ref: not Rural) | <0.001 (<0.001 - >999.99) | 0.9806 | -- | -- |
| Engagement with HF program | 1.817 (1.192 - 2.767) | 0.0055 | 1.639 (1.058 - 2.542) | 0.0271 |
| Body mass index (per kg/m^2^ increase) | 0.98 (0.953 - 1.006) | 0.1352 | 0.979 (0.953 - 1.007) | 0.1408 |
| Ischemic cardiomyopathy | 1.103 (0.728 - 1.673) | 0.6436 | -- | -- |
| Left ventricular ejection fraction (per % increase) | 0.997 (0.986 - 1.008) | 0.5812 | -- | -- |
| NYHA functional class III or IV (ref: I or II) | 1.065 (0.686 - 1.654) | 0.7799 | -- | -- |
| Recent diagnosis of heart failure within 18 months | 0.799 (0.544 - 1.175) | 0.2548 | -- | -- |
| Covid-19 positive | <0.001 (<0.001 - >999.999) | 0.9873 | -- | -- |
| **Coexisting conditions** | -- | -- | -- | -- |
| Atrial Fibrillation/Flutter | 1.030 (0.701 - 1.515) | 0.8793 | -- | -- |
| Diabetes | 1.315 (0.891 - 1.94) | 0.1681 | -- | -- |
| Dyslipidemia | 1.093 (0.743 - 1.607) | 0.6507 | -- | -- |
| Hypertension | 0.914 (0.599 - 1.396) | 0.6786 | -- | -- |
| Active smoking (ref: former or never) | 1.040 (0.492 - 2.199) | 0.9185 | -- | -- |
| Chronic obstructive pulmonary disease | 1.023 (0.612 - 1.710) | 0.9295 | -- | -- |
| Liver Cirrhosis | 1.923 (0.796 - 4.646) | 0.1461 | -- | -- |
| Cancer | 1.000 (0.641 - 1.560) | 1.0000 | -- | -- |
| Use of Dialysis | 0.691 (0.164 - 2.910) | 0.6145 | -- | -- |
| Dementia | 1.118 (0.567 - 2.202) | 0.7478 | -- | -- |
| Peripheral Vascular Disease | 1.801 (1.099 - 2.952) | 0.0196 | 1.683 (1.018 - 2.780) | 0.0422 |
| Cerebrovascular Accident = | 1.394 (0.839 - 2.317) | 0.1998 | -- | -- |
| Full code status | 0.842 (0.570 - 1.243) | 0.3875 | -- | -- |
| **Clinical measurements on initial presentation** | -- | -- | -- | -- |
| Systolic blood pressure (per mmHg increase) | 0.996 (0.988 - 1.003) | 0.2725 | -- | -- |
| Heart rate (per bpm increase) | 0.998 (0.989 - 1.006) | 0.6117 | -- | -- |
| Respiratory rate (per bpm increase) | 0.995 (0.962 - 1.030) | 0.7779 | -- | -- |
| QRS duration (per ms increase) | 1.001 (0.995 - 1.006) | 0.7514 | -- | -- |
| **Laboratory values** | -- | -- | -- | -- |
| Hemoglobin (per g/L increase) | 0.996 (0.988 - 1.004) | 0.3432 | -- | -- |
| Sodium (per meq/L increase) | 1.005 (0.984 - 1.026) | 0.6587 | -- | -- |
| Creatinine (per µmol/dL increase) | 1.000 (0.999 - 1.002) | 0.5835 | -- | -- |
| **Heart failure medication** | -- | -- | -- | -- |
| Beta-blocker | 1.230 (0.807 - 1.875) | 0.3354 | -- | -- |
| ACEi, ARB, or ARNi | 0.919 (0.623 - 1.356) | 0.6699 | -- | -- |
| Mineralocorticoid receptor antagonist | 1.449 (0.934 - 2.246) | 0.0976 | -- | -- |
| Implantable cardioverter defibrillator | 1.947 (1.164 - 3.258) | 0.0112 | 1.579 (0.906 - 2.751) | 0.1072 |
| Cardiac resynchronization therapy | 0.934 (0.332 - 2.633) | 0.8980 | -- | -- |
| Dose of Furosemide | 1.001 (0.999 - 1.004) | 0.3529 | -- | -- |

ACEi: angiotensin converting enzyme inhibitors; ARB: angiotensin receptor blockers; ARNi: angiotensin receptor-neprilysin inhibitor; CI: confidence interval; ED: emergency department; OR: odds ratio
